# CHAracteristics of research studies that iNfluence practice: a GEneral survey of Canadian orthopaedic Surgeons (CHANGES): a pilot survey

**DOI:** 10.1186/s40064-015-0855-4

**Published:** 2015-02-05

**Authors:** Darren de SA, Patrick Thornley, Nathan Evaniew, Kim Madden, Mohit Bhandari, Michelle Ghert

**Affiliations:** Division of Orthopaedic Surgery, Department of Surgery, McMaster University, HHS Hamilton General Hospital, 237 Barton Street, Hamilton, ON Canada L8L 2X2; Faculty of Health Sciences, Michael G. DeGroote School of Medicine, McMaster University, Hamilton, ON Canada; Department of Clinical Epidemiology and Biostatistics, McMaster University, Hamilton, Ontario Canada

**Keywords:** Evidence-based medicine, Orthopaedic surgery, Clinical practice, Patient care

## Abstract

**Background:**

Evidence Based Medicine (EBM) is increasingly being applied to inform clinical decision-making in orthopaedic surgery. Despite the promotion of EBM in Orthopaedic Surgery, the adoption of results from high quality clinical research seems highly unpredictable and does not appear to be driven strictly by randomized trial data. The objective of this study was to pilot a survey to determine if we could identify surgeon opinions on the characteristics of research studies that are perceived as being most likely to influence clinical decision-making among orthopaedic surgeons in Canada.

**Methods:**

A 28-question electronic survey was distributed to active members of the Canadian Orthopaedic Association (COA) over a period of 11 weeks. The questionnaire sought to analyze the influence of both extrinsic and intrinsic characteristics of research studies and their potential to influence practice patterns. Extrinsic factors included the perceived journal quality and investigator profiles, economic impact, peer/patient/industry influence and individual surgeon residency/fellowship training experiences. Intrinsic factors included study design, sample size, and outcomes reported. Descriptive statistics are provided.

**Results:**

Of the 109 members of the COA who opened the survey, 95 (87%) completed the survey in its entirety. The overall response rate was 11% (95/841). Surgeons achieved consensus on the influence of three key designs on their practices: 1) randomized controlled trials 94 (99%), 2) meta-analysis 83 (87%), and 3) systematic reviews 81 (85%). Sixty-seven percent of surgeons agreed that studies with sample sizes of 101–500 or more were more likely to influence clinical practice than smaller studies (n = <100). Factors other than design influencing adoption included 1) reputation of the investigators (99%) and 2) perceived quality of the journal (75%).

**Conclusion:**

Although study design and sample size (i.e. minimum of 100 patients) have some influence on clinical decision making, surgeon respondents are equally influenced by investigator reputation and perceived journal quality. At present, continued emphasis on the generation of large, methodologically sound clinical trials remains paramount to translating research findings to clinical practice changes. Specific to this pilot survey, strategies to solicit more widespread responses will be pursued.

## Introduction

There is an increasing awareness and application of evidence-based medicine (EBM) to guide clinical practice and enhance patient care in many medical and surgical specialties - and orthopaedic surgery is no exception (Dijkman et al. 2010; Panesar et al. 2010). Accordingly, forums such as journal clubs and society meetings, aimed at equipping the next generation of practitioners with skills to critically appraise the literature, are becoming more commonplace (Dirschl et al. 2003). Although it is clear how and why studies of low quality fail to influence clinical practice, what remains a conundrum is the lack of practice changes in the setting of high quality evidence.

“High-quality evidence”, often cited as the requirement for orthopaedic surgeons to consider changing clinical practice (Khan et al. 2013), is not always successful in effecting changes. Dijkman *et al.* demonstrated that only 62% of surgeons would change their practice if a multi-center, randomized-controlled trial (RCT) demonstrated more favourable outcomes from the studied intervention over their conventional method (Dijkman et al. 2010). In addition, Matzon *et al.* (Matzon et al. 2013) demonstrated poor adherence (less than 50%) to the American Society for Surgery of the Hand (ASSH) clinical practice guidelines regarding upper-extremity management among 469 members of the American Academy of Orthopaedic Surgeons (AAOS) (Matzon et al. 2013). While previous reports suggest that 90% of surgeons used RCTs to guide practice (Matzon et al. 2013), that number is much lower in orthopaedic surgery - with only 25% and 46% of surgeons finding RCTs “extremely” and “somewhat” helpful, respectively (Khan et al. 2013). The reasons for this are likely multi-factorial, with studies demonstrating surgeon preference (LeBlanc et al. 2014), increasing patient education/pressure (McKinlay et al. 2014), and challenges unique to practice setting and resource allocation (Farias-Kovac et al. 2014) as critical in determining patient treatment – independent of sound evidence. Moreover, specialty-specific differences in training and decision-making (Fraenkel et al. 2013) and/or the nature of the results (i.e. positive or negative) (Schwenk et al. 2002) may also affect practice change decisions. Thus, in order to guide the design and conduct of future studies each of these factors deserves further investigation.

The objective of this study was to pilot a survey to determine if we could identify surgeon opinions on which characteristics of research studies, both extrinsic and intrinsic, are perceived as being most likely to influence clinical decision-making among active orthopaedic surgeons in Canada.

## Materials & methodology

### Survey development

A focus group and detailed literature search were utilized to generate a novel instrument that assessed the study objectives while minimizing respondent burden. The focus group consisted of an orthopaedic surgeon experienced in conducting clinical research, a graduate student studying clinical epidemiology, and a junior resident in orthopaedic surgery. The questionnaire was designed and developed based on previously established guidelines (Burns et al. 2008; Eysenbach 2004). Emphasis was placed on ensuring the question stems were easy to understand/interpret. This required presenting them in an unbiased, nonjudgmental tone, and utilizing neutral language demonstrating cultural competence. Furthermore, the focus group assessed the visual appearance of the questionnaire to determine the most readable font type and size. A status bar was included throughout the questionnaire to help increase response rates (Burns et al. 2008). The final survey was reviewed by an additional expert in research methodology for face and content validity.

The survey was divided into a seven-question demographics section and a twenty-one-question respondent preferences section assessing what information would be important or unimportant in influencing changes in clinical practice. The demographic survey section contained questions about age and gender of the respondent, in addition to such information as subspecialty training status (where applicable), clinical research degrees, and current teaching status. We examined such extrinsic factors (i.e. those unrelated to the study methodology) as the perceived journal quality for publication and investigator profiles, economic impact, peer/patient/industry influence and individual surgeon residency/fellowship training experiences. Intrinsic factors (i.e. those related to study methodology and conduct) included study design, sample size, and outcomes reported. A print copy of the survey is available herein within the Appendix. The cover letter addressed the study objective, confidentiality, approximate time for completion, and contact data to obtain further information.

### Piloting

For ease of online survey administration to a busy study participant population, the survey was administered using SurveyMonkey® (www.surveymonkey.com, Palo Alto, CA). The final item list and electronic interface were pilot tested as it would appear to all respondents, across an independent group representing a sample of eight potential respondents (four surgeons in academic settings and four in community practices). This aimed to optimize the balance between information gathering and respondent burden in addition to assessing usability and technical functionality of the electronic survey interface (Burns et al. 2008; Eysenbach 2004). Furthermore, the pilot testing aided in refining the questionnaire for any question prompts that could potentially be misinterpreted.

### Questionnaire administration

Our population of interest was all practicing orthopaedic surgeons and our convenience-sampling frame was all 841 active members of the Canadian Orthopaedic Association (COA). The inclusion criteria were that respondents must have: 1) completed training in an approved program in orthopaedic surgery; 2) hold valid certification by the Royal College of Physicians and Surgeons of Canada or equivalent; 3) be listed as members of the COA; and 4) currently practice full-time orthopaedic surgery in Canada or the United States of America.

Requests for participation were sent via two batch e-mail communications from the COA headquarters. On April 24, 2014, the first e-mail included a cover letter on departmental letterhead and a secure link to complete the web-based questionnaire. Passive consent was implied as participants independently volunteered to complete the survey. No monetary incentives or pre-notification of the survey were provided. All responses were considered anonymous. A reminder e-mail was sent on June 27, 2014. Data collection concluded approximately 11 weeks post-launch on July 11, 2014.

Participants were asked to anonymously respond to all questions within the survey, which was programmed to only permit surveys completed in their entirety to be submitted through the online server.

This study and the questionnaire were approved by the Hamilton Integrated Research Ethics Board (Project Number: 14–169).

### Statistical analysis

Completed responses were entered into a study-specific database and data were analyzed descriptively. Incomplete responses were discarded. Categorical variables are reported as counts and percentages, and continuous variables are summarized with means and standard deviations (SD). Response rate was defined as the number of individuals who completed the survey across those who had been sent the e-mail invitation. The analyses were performed using IBM SPSS version 21 (IBM, Chicago, IL, USA, 2012) and Microsoft Excel (Microsoft, Santa Rosa, CA, USA, 2008).

## Results

### Demographics

The initial e-mail asking for respondent participation was sent out to the 841 active COA members with a listed e-mail address. Of the 841 to receive the first e-mail, 424 (50%) opened the e-mail, with 61 of these individuals (14%) opening the survey link. The second e-mail was again sent to the 841 active members of the COA with listed e-mail addresses, of which, 346 individuals opened the e-mail, with 48 of them (14%), opening the provided survey link. Of the 109 members of the COA to open the survey link, 95 (87%) completed the survey in its entirety. Thus, the overall response rate was 11%.

Eighty-five percent (81) of respondents were male (Table 1). Respondents have been actively practicing for a mean 16.4 years (SD 11.8 years), and 74% (70) reported supervising residents. Only 12% (11) of respondents possessed a graduate degree in clinical research. The top three fellowships represented in decreasing order were: Hip and Knee Reconstruction (35%), Trauma (23%), and Upper Extremity (20%). The majority of respondents 86% (82) agreed or strongly agreed that the judicious integration of best-available research with patient values and clinical expertise was an important part of their clinical decision-making (Table 1).

**Table 1:** **Demographics for canadian orthopaedic association (COA) respondents**

**Total responses: 95**	**No. (%) of respondents**
**Age**	Mean 48.8 years old (SD 11.3)
**Gender**	
**Male**	81 (85)
**Female**	14 (15)
**Number of years in practice**	Mean 16.4 years (SD 11.8)
**I am currently supervising residents**	
**Yes**	70 (74)
**No**	25 (26)
**I obtained subspecialty fellowship training in the following**	
**discipline**	
**Trauma**	22 (23)
**Hip and knee reconstruction/Total joint reconstruction**	33 (35)
**Upper extremity**	19 (20)
**Sports medicine**	16 (17)
**Spine**	11 (12)
**Foot and Ankle**	5 (5)
**Pediatrics**	8 (8)
**Oncology**	5 (5)
**Other**	5 (5)
**None**	13 (14)
**I hold a graduate degree in clinical research**	
**Yes**	11 (12)
**No**	84 (88)

### Extrinsic factors of research studies affecting clinical decision making

The reputation of both the study investigators and journal of publication appear to influence whether or not a surgeon is likely to implement given evidence into their clinical practice. 98 (Ninety-eight percent) respondents at least somewhat agreed that research published by a highly skilled subspecialist would likely influence clinical decision-making, with 59% noting that the study investigator profile was ‘important’ to ‘very important’. Ninety-seven percent of surgeons at least ‘somewhat’ agreed that investigator profile was significant even for unpublished research presented at a conference. Where one published material is of comparable influence, with 75% of respondents valuing the perceived quality of the journal in which the study is published as ‘important’ to ‘very important’ in their clinical decision-making, and 97% ‘somewhat’ to ‘strongly’ agreeing that research from highly prestigious journals was likely to influence practice changes (Table 2).

**Table 2:** **Extrinsic factors of research studies affecting clinical decision making**

**Item asked**	**No. (%) of respondents**
**The judicious integration of best-available research with patient values and clinical expertise is an important part of my clinical decision-making**	
Strongly Agree	39 (41)
Agree	43 (45)
Somewhat Agree	13 (14)
Somewhat Disagree	0 (0)
Disagree	0 (0)
Strongly Disagree	0 (0)
**Research published in a highly prestigious journal is likely to influence my clinical decision-making**	
Strongly Agree	9 (9.5)
Agree	42 (44)
Somewhat Agree	41 (43)
Somewhat Disagree	1 (1.1)
Disagree	2 (2.1)
Strongly Disagree	0 (0)
**Research published by highly skilled subspecialist surgeons is likely to influence my clinical decision-making**	
Strongly Agree	6 (6.3)
Agree	44 (46)
Somewhat Agree	43 (45)
Somewhat Disagree	0 (0)
Disagree	2 (2.1)
Strongly Disagree	0 (0)
**Research presented at a conference by highly skilled subspecialist surgeons is likely to influence my clinical decision-making**	
Strongly Agree	8 (8.4)
Agree	40 (42)
Somewhat Agree	44 (46)
Somewhat Disagree	0 (0)
Disagree	3 (3.2)
Strongly Disagree	0 (0)
**The opinions of my direct colleagues are likely to influence my clinical decision-making**	
Strongly Agree	6 (6.3)
Agree	35 (37)
Somewhat Agree	49 (52)
Somewhat Disagree	0 (0)
Disagree	5 (5.3)
Strongly Disagree	0 (0)
**Information that I received during my fellowship training is likely to influence my clinical decision-making**	
Strongly Agree	21 (22)
Agree	44 (46)
Somewhat Agree	44 (46)
Somewhat Disagree	29 (31)
Disagree	0 (0)
Strongly Disagree	1 (1.1)
	0 (0)
**Information that I received during my residency is likely to influence my clinical decision-making**	
Strongly Agree	13 (14)
Agree	36 (38)
Somewhat Agree	45 (48)
Somewhat Disagree	0 (0)
Disagree	1 (1.1)
Strongly Disagree	0 (0)
**Direct company-to-patient advertising is likely to influence my clinical decision-making**	
Strongly Agree	0 (0)
Agree	0 (0)
Somewhat Agree	11 (12)
Somewhat Disagree	9 (9.5)
Disagree	51 (54)
Strongly Disagree	24 (25)
**Pressure from patients is likely to influence my clinical decision-making**	
Strongly Agree	0 (0)
Agree	0 (0)
Somewhat Agree	17 (18)
Somewhat Disagree	17 (18)
Disagree	41 (43)
Strongly Disagree	20 (21)
**The economic impact of the proposed intervention is likely to influence my clinical decision-making**	
Strongly Agree	1 (1.1)
Agree	20 (21)
Somewhat Agree	55 (58)
Somewhat Disagree	3 (3.2)
Disagree	11 (12)
Strongly Disagree	5 (5.3)
**My surgical experience and personal preferences guide my practice more than research**	
Strongly Agree	5 (5.3)
Agree	19 (20)
Somewhat Agree	47 (50)
Somewhat Disagree	5 (5.3)
Disagree	17 (18)
Strongly Disagree	2 (2.1)
**I am likely to continue with the practices I developed during my training and currently use, independent of new research**	
Strongly Agree	0 (0)
Agree	9 (9.5)
Somewhat Agree	14 (15)
Somewhat Disagree	14 (15)
Disagree	43 (45)
Strongly Disagree	15 (16)
**Independent of literature, I will most likely implement a proposed intervention that appears harmless to patients**	
Strongly Agree	3 (3.2)
Agree	9 (9.5)
Somewhat Agree	28 (30)
Somewhat Disagree	12 (13)
Disagree	36 (38)
Strongly Disagree	7 (7.4)

Only 12% and 18% of surgeons reported being ‘somewhat’ to ‘strongly’ likely to be influenced by direct company-to-patient advertising and pressure from patients, respectively, and though the economic impact of a proposed intervention is ‘somewhat’ to ‘strongly’ likely to influence practice changes in 80% of respondents, less than half (42%) reported being likely to implement a proposed intervention that appears harmless to patients, independent of the literature (Table 2).

The surgeon respondents appear to be receptive to evidence in their clinical decision-making. Although 75% of surgeons are ‘somewhat’ to ‘strongly’ guided by personal experience and preferences over research - with 99% ‘somewhat’ to ‘strongly’ influenced by both their residency and fellowship training - 76% would likely not continue with these practices in light of new research favouring alternatives. Much less emphasis is held in the opinions of direct colleagues, with 52% believing this would influence their clinical decision-making (Table 2).

### Intrinsic factors of research studies affecting clinical decision making

Almost all surgeons (98%) reported that the study design was an ‘important’ to ‘very important’ factor likely to influence their clinical decision-making. The top three designs with the potential to influence a change in clinical practice were: randomized control trials (99%), meta-analysis (87%), and systematic reviews (85%). Moreover, systematic review (84%) and narrative review (48%) designs were felt by respondents to have had the most profound impact on their practice in the last five years, with cohort (5%), case control (5%), case series (2%), and case report (5%) designs having minimal influence. Sample size was reported as being an ‘important’ to ‘very important’ factor for 95% of respondents, with 67% feeling that examining a sample size of 101 to 500 would be necessary to influence clinical practice (Table 3).

**Table 3:** **Intrinsic factors of research studies affecting clinical decision making**

**Item asked**	**No. (%) of respondents**
**Perceived quality of the journal:**	
Very Important	17 (18)
Important	54 (57)
Somewhat Important	21 (22)
Not at all Important	3 (3.2)
**Profile of the study investigators:**	
Very Important	10 (11)
Important	46 (48)
Somewhat Important	29 (31)
Not at all Important	10 (11)
**Study design:**	
Very Important	60 (63)
Important	33 (35)
Somewhat Important	2 (2.1)
Not at all Important	0 (0)
**Study sample size:**	
Very Important	47 (50)
Important	43 (45)
Somewhat Important	4 (4.2)
Not at all Important	1 (1.1)

In terms of outcomes reporting, 82% and 78% of respondents felt that a study should report both p-values and 95% confidence intervals, respectively. A key finding is that only 18% and 30% of respondents required a number needed to treat (NNT) and a minimal important difference (MID) respectively - both outcomes with direct clinical applicability - as an outcome that would influence their clinical practice (Table 4).

**Table 4:** **Intrinsic factors of research studies affecting clinical decision making**

**Item asked**	**No. (%) of respondents**
**The results of the following study designs have the potential to influence a change in my clinical practice (select all that apply to you):**	
Meta-Analysis	83 (87)
Systematic Review	81 (85)
Randomized Control Trial	94 (99)
Cohort Study	40 (42)
Case-Control Study	47 (50)
Cross-Sectional Study	21 (22)
Case Series	22 (23)
Case Report	9 (9.5)
Narrative Review	8 (8.4)
Editorial	11 (12)
**For a study to influence my clinical practice it would require a sample size of (select all that apply to you):**	
11-50	24 (25)
51-100	45 (47)
101-500	64 (67)
501-1000	36 (38)
>1001	35 (37)
**For a study to influence my clinical practice the results should report (select all that apply to you):**	
P Value	78 (82)
95% Confidence Interval	74 (78)
Relative Risk Reduction	48 (51)
Absolute Risk Reduction	36 (38)
Odds Ratio	37 (39)
Number Needed to Treat	17 (18)
Mean Difference	35 (37)
Minimally Important Difference	28 (30)
Sensitivity Analysis	14 (15)
Adjusted Analysis	4 (4.2)
None of the Above	42 (44)
**The study design that has had the most profound impact on my practice in the last five years is (select all that apply to you):**	
Meta-Analysis	9 (9.5)
Systematic Review	80 (85)
Randomized Control Trial	14 (15)
Cohort Study	5 (5.3)
Case-Control Study	5 (5.3)
Cross-Sectional Study	2 (2.1)
Case Series	2 (2.1)
Case Report	5 (5.3)
Narrative Review	46 (49)
Editorial	0 (0)

## Discussion

### Key findings

Evidence-based orthopaedics is aimed at integrating sound research with surgeon expertise and patient values (Schemitsch et al. 2009), and 86% of survey respondents agreed that incorporating sound evidence plays a major role in their practice decisions. The objective of this pilot survey project was to determine which characteristics of research studies, both extrinsic and intrinsic, are perceived as being most likely to influence actual clinical decision-making among orthopaedic surgeons in Canada.

In this survey of 95 active members of the Canadian Orthopaedic Association (COA), our findings suggest that both specific extrinsic and intrinsic elements of a research study exist that surgeons consider key features for potentially influencing practice changes. Namely, surgeons currently in practice value both the investigator profile/reputation producing the research, as well as the perceived quality of the journal in which it is published as key influential features - and are not persuaded by direct company-to-patient advertising or patient pressure. Within a given study, surgeons are more likely to apply the evidence to their practice if the design is amongst the highest quality (i.e. meta-analyses, systematic reviews, randomized controlled trials), if sample sizes range from 101–500 subjects, and if p-values with associated 95% confidence intervals are reported. However, only 18% and 30% of respondents required a number needed to treat (NNT) and a minimal important difference (MID), respectively, as an outcome measure that would likely influence practice changes.

### Strengths and limitations

Strengths of this study encompass the in-depth efforts in designing and implementing a meaningful and user-friendly questionnaire to arrive at an optimal balance between information gathering and respondent burden. Our efforts to pilot the study and establish face and content validity strengthen our findings. We were able to ensure respondent anonymity, secure data collection, and provide appropriate time estimates for completion as per well established guidelines - all limiting the chance of incomplete surveys (Burns et al. 2008). This is also the first study, to our knowledge, that has sought the opinions of practicing surgeons with regards to features of a research design deemed valuable in guiding clinical practice. However, surgeon “opinion” may not necessarily translate into surgeon “action”. It is possible that how one responds to a survey may be different from how one acts clinically. Nevertheless, the anonymity of the current survey administration is a first and critical step to ascertaining true surgeon attitudes, which in turn can serve as a springboard for future objective studies validating actual practice changes.

Similarly, this study aimed to ascertain any and all factors deemed valuable in applying evidence to clinical practice, in light of the hypothesis that such transition is likely multi-factorial. Using our Likert scale, efforts were made to determine how important an individual factor may be to a surgeon, but caution should be exercised not to interpret our data to mean that all intrinsic and extrinsic factors examined were equal in their potential influence. This would be more appropriately answered by asking respondents to rate or rank order each factor, which was not done here and is arguably a limitation that can be an area examined in future research.

This study primarily addressed the attitudes of Canadian surgeons, and therefore, future efforts aimed at assessing whether these results are germane to surgeons outside of Canada would be of interest. There exists a possibility that certain factors not identified by Canadian surgeons or analyzed in this study exist outside Canada, with an undefined influence on practice. Similarly, due to inherent political, legal, and cultural differences, even factors that exist within Canada such as industry influence, litigation risks, and access to implants for example, may have different impacts in other countries, and thus, may result in varying surgeon opinions and preferences in guiding clinical practice.

Our response rate of 11% is an important limitation and a weak point of this pilot attempt to answer our research question. Typically, only 51% of surveys report a response rate (Rosen and Olsen 2006). Our low survey response rate of 11% (95/841), and higher proportion of academic surgeons (74%) may introduce selection bias and impact generalizability. However, 841 represents the total number of active members of the COA, not all of whom provide e-mail addresses with their membership application. Moreover, of those that do provide an e-mail address, exactly how many provide personal versus office addresses is unknown. Even amongst those that did receive an e-mail, not all had opened it, and of those who did, not all accessed the survey link. Arguably, the 11% response rate and majority academic surgeons may not truly reflect the thinking of the entire COA but is comparable to several recent survey publications surveying COA members (Abouali et al. 2013; Almeida 2000; Khan et al. 2013; Pally and Kreder 2013; Tam et al. 2006). This is also consistent with response rates traditionally from surveys on surgeons, which range from 15-27% (Almeida 2000; Khalily et al. 2000; Matarasso et al. 2000).

One area that may have improved response rates would have been to develop and administer the survey in French in addition to English, via mail, and/or in-person at the COA Annual Meeting. We know from personal communication with our contact from COA headquarters that 135 active members identified French as their first language. How significant English-only versus English and French surveys are to increasing response rates in Canada remains unknown. As per Bhandari *et al.* (Bhandari et al. 2008), current estimates of the COA suggest that >80% of practicing orthopaedic surgeons in Canada are members and that 91% are men. We surveyed active members of the COA independent of practice location, and as Table 1 demonstrates, have a sufficient breadth in terms of surgeon age, length of practice, and fellowship training. Other general orthopaedic surgeon demographic statistics (i.e. Academic versus Community practice) of those non-responders or the COA membership as a whole (i.e. how many practising orthopaedic surgeons are not COA members) were not available for comparison.

Interestingly, although study design was universally an ‘important’ to ‘very important’ factor for likely influencing clinical practice, with 99% of respondents reporting that RCT study designs have the potential to influence a change in clinical practice, only 15% noted that RCTs had a profound impact on their practice in the past five years. In fact, Bhandari *et al.* (Schemitsch et al. 2009) reported in a 2007 survey of members of the American Orthopaedic Association that 82% believed that RCTs were not able to answer the majority of important clinical and research questions, citing etiologic, incidence and prognostic-aimed studies as examples not appropriate for RCT. Therefore, it was surprising that other study designs such as cohort, case control, case series, and even case reports had minimal impact on the practice changes of the current respondents in the past five years. Certainly, these designs comprise the majority of patient-directed study designs in orthopaedics, and with increased reliance on systematic and narrative reviews, discerning what elements of these designs appeal to practicing surgeons warrants further investigation. With surgeons more likely to be influenced by systematic review designs, there has been a resultant increase in the number of systematic reviews published, independent of subspecialty (Bhandari et al. 2004b; Bhandari et al. 2001; Moher et al. 2007).

Our findings highlight the disconnect between what surgeons currently value in published research and the recent move towards evidence-based orthopaedics. Active surgeons appeared to value both the perceived quality of the journal and the investigator profile when considering changing clinical practice. However, the perceived journal quality, often assessed by an Impact Factor, does not necessarily correlate with study quality or quality of reporting, is subject to self-citation bias, and thus, should be interpreted with caution (Gluud et al. 2005; Siebelt et al. 2010; Theodoropoulos et al. 2012). It has not escaped notice that using investigator profile as a major criterion is problematic, given that perceptions vary amongst readers regarding author contributions and authorship position - often causing readers to draw false conclusions about author credit (Bhandari et al. 2004a; Bhandari et al. 2003; Bhandari et al. 2014; Tornetta et al. 2009). Moreover, having investigator profile as a significant factor in whether or not research translates to clinical practice is vulnerable to the issues with “ghost” authorship - that is, individuals who significantly contribute to the production of a manuscript, but that are not cited as authors, potentially masking conflicts of interest.

Currently, the literature in Orthopaedic Surgery is expanding at approximately 4,000 articles across 100 journals per month (OrthoEvidence 2014). An orthopaedic surgeon in practice would need to read and evaluate 17 articles per day to remain current (OrthoEvidence 2014) and advance their expertise. The glaring discrepancy between what surgeons currently utilize to translate knowledge into practice from what they should be utilizing may reflect a general lack of knowledge about research methodology. Thus, changes are required, and the focus must be both on further educating current surgeons and trainees, and increasing efforts to make available pre-appraised, evidence summaries of the best available literature. Given that high quality large RCTs remain highly endorsed with regards to influencing changes of practice, we wish to encourage practicing Orthopaedic Surgeons to consult OrthoEvidence (www.myorthoevidence.com) to access a large repository of high quality randomized trials and systematic reviews in orthopaedics for ‘actionable’ data applicable to daily practice.
